# Efficacy and safety of percutaneous renal biopsy performed using 18G needle versus 16G needle: a single-center retrospective study

**DOI:** 10.1007/s11255-022-03276-4

**Published:** 2022-07-04

**Authors:** Senyin Xu, Lili Ma, Jiazhen Lin, Zhengxian Zhang, Xiaoya Wang, Jiazhen Yin

**Affiliations:** 1 Department of Ultrasound, Zhejiang Hospital, Hangzhou, Zhejiang China; 2 Department of Ultrasound, Hangzhou TCM Hospital Affiliated to Zhejiang Chinese Medical University, Hangzhou, Zhejiang China; 3 Zhejiang Chinese Medical University, Hangzhou, Zhejiang China; 4 Department of Nephrology (Key Laboratory of Management of Kidney Disease in Zhejiang Province), Hangzhou TCM Hospital Affiliated to Zhejiang Chinese Medical University, Hangzhou, Zhejiang China

**Keywords:** Percutaneous, Renal biopsy, Needles, Complication, Adequacy

## Abstract

**Background:**

At present, both 16G and 18G needles are used for percutaneous renal biopsy in China. This study aimed to compare the efficacy and safety of biopsy performed with the 18G needle vs. the 16G needle.

**Methods:**

The data of patients who underwent percutaneous renal biopsy at our hospital between January 2015 and December 2019 were retrospectively analyzed. The number of glomeruli obtained by puncture and postoperative complications were compared between patients undergoing biopsy with the 16G and 18G needles. Continuous variables were compared by the t test or the Mann–Whitney *U* test, and categorical variables by the chi-square test. Correlation analysis was used to examine the relationship of different variables with hematoma size.

**Results:**

Of the total 3138 kidney biopsies, 2526 were performed with the18G needle and 612 with the 16G needle. The number of glomeruli obtained was not significantly different between the two groups (*P* = 0.078). Large hematomas were significantly more common the 16G group than in the 18G group (9.31% vs. 5.98%, *P* = 0.003). Arteriovenous fistula was also more common in the 16G group (1.14% vs. 0.23%, *P* = 0.005). Other complications were rare, with similar incidence in the two groups.

**Conclusion:**

The 18G needle is as effective as the 16G needle for percutaneous renal biopsy. The risk of large hematoma and arteriovenous fistula appear to be lower with the 18G needle.

## Introduction

Percutaneous renal biopsy (PRB) has been used since the early 1950s and has now become the gold standard for diagnosis of kidney pathology [1]. The gauge of the needle used for PRB varies from 14 to 22G [2–5]. In the US, the 16G needle is recommended as it is believed that this gauge is ideal for obtaining sufficient tissue, with minimal bleeding complications [6]; no similar recommendations have been made in China.

The safety and success of PRB have improved markedly with the introduction of semiautomatic biopsy needles and real-time ultrasound guidance [7–9]. Most centers now use semiautomatic biopsy needles, the popular choices being the 16G needles (diameter 1.65 mm, length 22 mm or 15 mm) and the 18G needles (diameter 1.27 mm, length 22 mm or 15 mm). The smaller 18G needle is usually preferred for patients with relative contraindications such as renal insufficiency, coagulation dysfunction, and small kidney size (length ≤ 9 cm) [10]. However, there is still some controversy about whether the 18G biopsy needle can obtain sufficient tissue for disease diagnosis or significantly reduce the incidence of complications.

The purpose of this retrospective study was to compare the efficacy and safety of PRB performed using the 18G needle versus the 16G needle.

## Materials and methods

### Patients

The data of 3138 patients who underwent PRB in our hospital between January 2015 and December 2019 were retrospectively analyzed. At our center, during 2015 and most of 2016, both 16G and 18G needles (CR Bard Inc., Tempe, AZ, USA) were used for PRB. The 18G needle was preferred for patients with relative contraindication such as renal insufficiency, coagulation disorder, and small kidney size, and the 16G needle for the other patients. However, after October 2016, only 18G needles were purchased, and so all biopsies were performed with 18G needle. Since 2017, only one type of renal puncture needle, that is, the 18G puncture needle, has been available at our center. This data analysis was conducted to analyze whether the use of 18G puncture needles causes a decrease in the number of specimens and whether there is an improvement in the risk of bleeding.

Thus, the patients could be divided into two groups: the 16G group and the 18G group. Safety and efficacy were compared between the two groups.

This study was a retrospective study that was approved by the hospital ethics committee (approval number 2020KY041). All patients provided informed consent for the renal biopsy. Informed consent from a parent and/or legal guardian was obtained for minors.

### Renal biopsy procedure

Prior to the biopsy all patients underwent complete kidney ultrasound examination, blood routine examination, liver and kidney function tests, and blood coagulation function tests. Anticoagulants (including aspirin, warfarin, heparin, and direct factor Xa inhibitors) were stopped 3–7 days before the procedure.

**Fig. 1:**
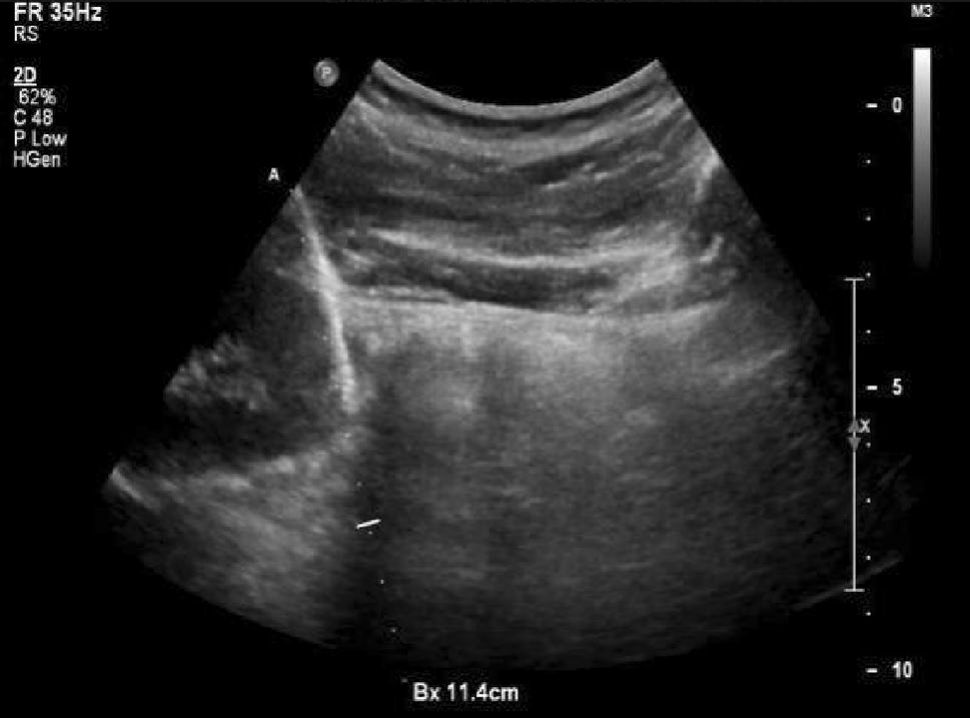
Ultrasound-guided line and puncturing route of kidney

The biopsy was performed by a nephrologist, with an ultrasonologist in attendance to assist in real-time ultrasound guidance [11]. The patient was asked to empty the bladder and lie in the prone position. The lower pole of the right kidney was selected as the biopsy target. The puncture site was disinfected and infiltrated with a local anesthetic. The guide needle was inserted and advanced till the tip reached the kidney capsule to trigger the biopsy needle and initiate the kidney biopsy (Fig. 1). Since sufficient tissue was necessary for light and electron microscopy, as well as immunofluorescent examination, two biopsy specimens were generally obtained; if the tissue was insufficient, up to five specimens were obtained. The biopsy tissue was divided into three parts and sent for examination.

**Fig. 2:**
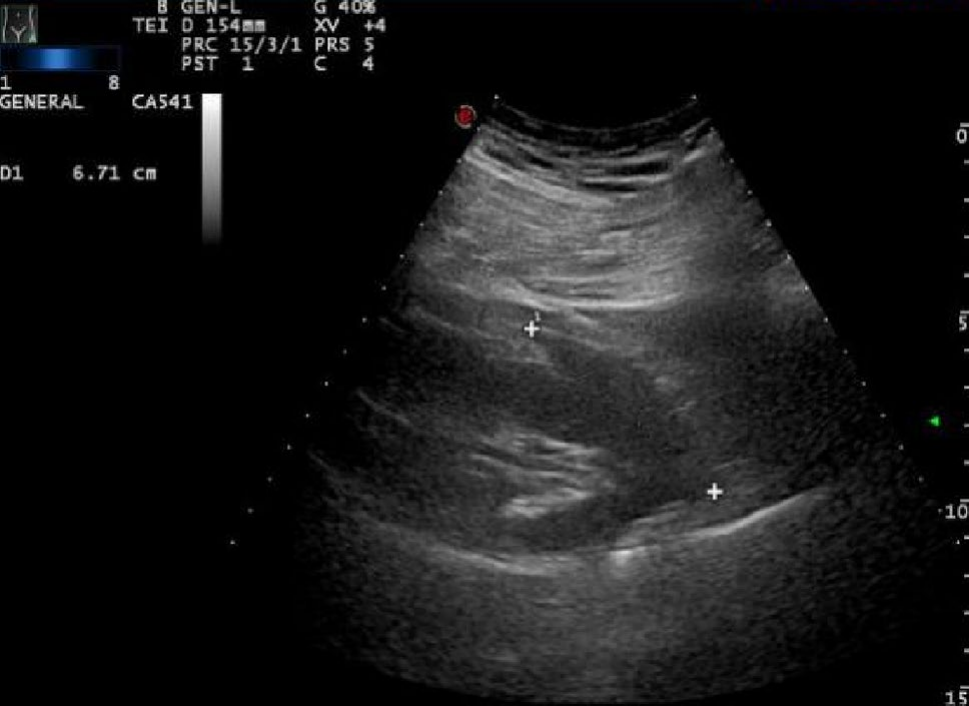
Length of hematoma after kidney puncture

The patient was allowed to assume the supine position 6–8 h after the procedure, and then rested in bed for 24 h. During this period, urine color and abdominal pain were recorded, and ultrasound examination was performed to detect formation of hematoma (Fig. 2). If gross hematuria appeared, immediate ultrasound examination was carried out; otherwise, ultrasound was performed 48–72 h after the procedure to assess the size of the renal subcapsular hematoma. The length, width, and height of the renal hematoma were measured, and the hematoma volume (V) was calculated using the formula [12].

$$ V = \pi /3 \times {\text{length}} \times {\text{width}} \times {\text{height}}. $$

If the hematoma volume was > 40 mL, regular (once every two days) ultrasound monitoring was carried out until the hematoma had completely disappeared or the hematoma volume was < 5 mL. If an arteriovenous fistula was found, ultrasound examination was repeated regularly (once every two weeks) until the arteriovenous fistula had disappeared.

## Observation indices

### Safety

Safety was assessed using the indices recommended by Whittier et al. [13, 14]: i.e., incidence of lumbar or abdominal pain, gross hematuria, arteriovenous fistula formation, bleeding requiring surgical intervention or blood transfusion, decreased blood pressure, decreased hemoglobin level, severe infection, and perirenal hematoma size.

### Efficacy

Efficacy of biopsy was assessed by counting the number of glomeruli obtained in the samples, with ≥ 10 glomeruli considered as an adequate sample. The adequate sample rate was compared between the two groups.

### Statistical analysis

The data were analyzed using SPSS 22 (IBM Corp., Armonk, NY, USA). Quantitative data were summarized as means (± standard deviation) or as medians (and interquartile range), depending on the normality of the distribution, and compared between groups using the independent samples t test or the Mann–Whitney *U* test, respectively. Qualitative data were summarized as percentages and compared between groups using the chi-square test. Linear correlation was used to analyze the correlation between the indexes, and the statistic was *R* (Spearman rank correlation was used if the condition was not met, and the statistic was RS). *P* < 0.05 was regarded as statistically significant.

## Results

The 3138 patients included in this study ranged in age from 10 to 79 years. The 18G group comprised 2526 patients with mean age of 41.98 ± 14.17 years, while the 16G group comprised 612 patients with mean age of 40.44 ± 14.29 years. Age was significantly higher in the 18G group (*P* = 0.015). Before biopsy, there were no significant differences in serum creatinine level, hemoglobin level, platelet count, and 24-h urine protein level between the two groups. The mean glomerular filtration rate (GFR) was significantly lower in the 18G group than in the 16G group: 83.44 ± 34.86 mL/min vs. 87.00 ± 37.25 mL/min, *P* = 0.026). The median cystatin C level was significantly higher in the 18G group than in the 16G group: 0.88 (0.70–1.22) mg/L vs. 0.82 (0.67–1.15) mg/L, *P* = 0.007 (Table 1).

**Table 1:** Baseline demographic and laboratory characteristics and size of punctured kidney in the two groups

	18G (*n* = 2526)	16G (*n* = 612)	*P*
Age, years	41.98 ± 14.17	40.44 ± 14.29	0.015*
Sex (male/female)	1149/1377	282/330	0.792
BMI (kg/m^2^)	22.83 (20.51–25.24)	23.18 (20.76–25.29)	0.257
Hemoglobin (g/L)	122.48 ± 21.69	124.24 ± 20.39	0.059
Platelet count (10^9^/L)	224.84 ± 68.27	220.46 ± 70.01	0.157
Serum creatinine (µmol/L)	75.0 (58.00–104.00)	73.00 (58.00–98.00)	0.107
GFR (mL/min)	83.44 ± 34.86	87.00 ± 37.25	0.026*
24-h urinary protein (g/d)	1.08 (0.48–2.65)	1.10 (0.45–2.90)	0.808
Cystatin C (mg/L)	0.88 (0.70–1.22)	0.82 (0.67–1.15)	0.007**
Kidney length before puncture (mm)	104.42 ± 8.57	104.74 ± 8.70	0.551
Kidney width before puncture (mm)	53.71 ± 5.00	53.90 ± 5.42	0.568
Kidney height before puncture (mm)	44.95 ± 5.28	45.23 ± 5.40	0.403
Parenchymal thickness of kidney before puncture (mm)	12.32 ± 1.32	12.29 ± 1.43	0.657

Values are the means ± standard deviation or the medians (interquartile range)

*BMI* body mass index, *GFR* glomerular filtration rate

*Statistically significant

### Efficacy

The efficacy of kidney biopsy is evaluated based on whether a sufficient number of glomeruli are obtained. The mean number of glomeruli obtained by renal puncture was comparable between the groups: 23.6 ± 10.1 in the 18G group vs. 22.8 ± 9.9 in the 16G group (*P* = 0.078). The number of glomeruli ≤ 9 cases is considered pathological glomerulus insufficient, which had 138 cases (5.4%) in 18G group and 34 cases (5.5%) in 16G group; there is no statistical difference between the two groups (*P* = 0.573). A sufficient number of glomeruli is considered to be ≥ 10. The number of glomeruli was ≥ 10 in 2388 cases (94.5%) in the 18G group and 578 cases (94.4%) in the 16G group (Table 2). There was no statistical difference between the two groups (*P* = 0.928).

**Table 2:** Comparison of the number of glomeruli obtained by use of the 18G and 16G needles

	18G (*n* = 2526)	16G (*n* = 612)	*P*
Mean number of glomeruli	23.59 ± 10.14	22.78 ± 9.93	0.078
≤ 4 glomeruli	21 (0.8%)	7 (1.1%)	0.624
5–9 glomeruli	117 (4.6%)	27 (4.4%	
10–19 glomeruli	823 (32.6%)	213 (34.8%)	
≥ 20 glomeruli	1565 (62.0%)	365 (59.6%)	

### Safety

The main complication after renal puncture was perirenal hematoma. Large hematomas (i.e., maximum diameter > 5 mm) occurred in 208/3138 (6.62%) patients. They were significantly less common in the 18G group than in the 16G group: 151/2526 (5.98%) vs. 57/612 (9.31%), *P* = 0.003. Other complications (including arteriovenous fistula formation, abdominal pain, gross hematuria, bleeding requiring blood transfusion, hemostasis requiring intervention, and death) were rare, with incidence < 1%. Arteriovenous fistula was significantly less common in the 18G group than in the 16G group: 6/2526 (0.23%) vs. 7/612 (1.14%), *P* = 0.005. Serious hemorrhage, requiring renal artery embolization to stop bleeding, was necessary in 1/2526 (0.04%) patient in 18G group. The incidence of other complications was similar in the two groups (Table 3). There were no deaths in either of the two groups.

**Table 3:** Complications after renal puncture in the two groups

Complication	18G (*n* = 2526)	16G (*n* = 612)	*P*
Large hematoma (≥ 50 mm)	151 (5.98%)	57 (9.31%)	0.003**
Arteriovenous fistula	6 (0.23%)	7 (1.14%)	0.005**
Abdominal pain	10 (0.39%)	3 (0.49%)	> 0.99
Gross hematuria	12 (0.48%)	6 (0.98%)	0.235
Blood transfusion	7 (0.28%)	3 (0.49%)	0.660
Surgical intervention for embolism or nephrectomy	1 (0.04%)	0	> 0.99
Death	0	0	

Since perirenal hematoma after renal puncture was the main complication, we compared hematoma volume between the two groups. The hematoma volume deviation was large, and the volume was calculated to analyze after log10 logarithm. As shown in Table 4, the length of the hematoma was 24.00 (18.00–33.00) mm in the 18G group vs. 26.00 (20.00–37.00) mm in the 16G group (*P* < 0.001). The height of the hematoma in the 18G group was 8.00 (6.00–11.00) mm, and the height of the hematoma in the 16G group was 10.00 (8.00–12.00) mm. There was a significant difference between the two groups (*P* < 0.001). The logarithm (log10) of the hematoma volume in the 18G group was 3.66 ± 0.54, and the logarithm (log10) of the hematoma volume in the 18G group was 3.80 ± 0.54, which had a significant difference between the two groups (*P* = 0.002). According to the classification of no hematoma, small hematoma (hematoma long diameter < 50 mm), large hematoma (hematoma long diameter ≥ 50 mm), there were 381 cases (15.1%) without hematoma and 151 cases (6.0%) with large hematoma in the 18G group, 68 cases (11.1%) without hematoma and 57 cases (9.3%) with large hematoma in the 16G group, with a significant difference between the two groups (*P* = 0.001).

**Table 4:** Comparison of hematoma size in the two groups

	18G	16G	*P*
Hematoma length, mm, median (IQR)	24.00 (18.00–33.00)	26.00 (20.00–37.00)	< 0.001
Hematoma width, mm, median (IQR)	9.00 (6.00–18.00)	9.00 (6.00–17.00)	0.069
Hematoma height, mm, median (IQR)	8.00 (6.00–11.00)	10.00 (8.00–12.00)	< 0.001
Log_10_ of hematoma volume, mean (± SD)	3.66 ± 0.54	3.80 ± 0.54	0.002
No hematoma, *n* (%)	381 (15.1%)	68 (11.1%)	0.001
Small hematoma (< 50 mm), *n* (%)	1994 (78.9%)	487 (79.6%)	
Large hematoma (> 50 mm), *n* (%)	151 (6.0%)	57 (9.3%)	

*IQR* interquartile range, *SD* standard deviation

We also attempted to identify the factors related to hematoma size. The scatter plot showed only a very weak correlation between age and hematoma size; the linear correlation coefficient ρ was 0.051. However, because these two indicators, especially the hematoma length, were non-normally distributed, Spearman rank correlation was used. As Table 5 shows, hematoma volume showed weak, but statistically significant, correlation with platelet count, serum creatinine, and cystatin C, all of which were weakly related.

**Table 5:** Analysis of factors related to hematoma volume

	Age (years)	Sex (male/female)	BMI (kg/m^2^)	Hemoglobin (g/L)	Platelet count (× 10^9^/L)	Serum creatinine (µmol/L)	GFR (mL/min)	24-h urinary protein (g/d)	Cystatin C (mg/L)	Kidney length (mm)
Hematoma volume related ρ	0.049	− 0.032	0.048	− 0.33	− 0.083	0.064	− 0.063	0.11	0.07	0.001
P value	0.116	0.297	0.130	0.294	0.008*	0.041*	0.44	0.73	0.025*	0.979

## Discussion

This retrospective study aimed to determine the efficacy and safety of the 18G needle for percutaneous renal biopsy. The ability to obtain sufficient glomeruli, with minimal bleeding, was compared between the 18G needle and the 16G needle. Although the current standards for the number of glomeruli necessary for diagnosis in autologous kidneys and transplanted kidneys are different [15, 16], it is generally believed that at least 10 glomeruli are required for light microscopic evaluation [17]. This study found that among the cases with glomerulus ≤ 9, there were 138 cases (5.4%) in the 18G group and 34 cases (5.5%) in the 16G group. There was no statistical difference between the two groups (*P* = 0.573). Moreover, the mean number of glomeruli obtained by renal puncture was similar in the 18G and 16G groups (23.59 ± 10.14 vs. 22.78 ± 9.93, *P* = 0.078). At present, the number of glomeruli is generally used to evaluate the success of renal puncture, and less than 10 glomeruli is considered as the evaluation standard. Based on this standard, the failure rates of the 18G and 16G needles were 5.5% and 5.4%, respectively, but the difference was not significant. At present, it is believed that good renal tissue samples contain more than 20 glomeruli. This criterion was met by 62.0% of the 18G needles and 59.6% of the 16G needles, respectively, but this difference was also not significant.

Previous studies have reported conflicting results regarding the efficacy of 18G needles; some studies showed that 16G needles were better than 18G needles for obtaining a sufficient number of glomeruli [10, 18], while others reported no difference between 18 and 16G needles [19, 20]. Based on the findings of the present study, we believe that there was no significant difference in efficacy between the two. However, as part of the results of the electron microscope were sent out for inspection, some of the fluorescent sections did not have any glomeruli, and then, the sections were resectioned from the wax blocks for immunohistochemical staining. Therefore, it was difficult to calculate the number of fluorescent and electron microscope spheres, so it might have an impact on the final number of the kidney. The method of cutting the light microscope and the experimental personnel are all fixed personnel, and the efficacy of renal puncture can only be evaluated from the number of small balls of the light microscope.

In this study, the main complications were perirenal hematoma and arteriovenous fistula. The risk of large hematoma and arteriovenous fistula were significantly lower with the smaller 18G needle. The overall incidence of large hematomas was 6.38%, with a significantly higher incidence in the 16G group than in the 18G group (9.31% vs. 5.98%, *P* = 0.003). The overall incidence of arteriovenous fistula was 0.04%, with significantly higher incidence in the 16G group than the 18G group (1.14% vs. 0.23%, *P* = 0.005). Large hematomas may cause abdominal pain and decrease blood hemoglobin level; some patients may require blood transfusion or even interventional hemostasis. Through our research, we believe that the thickness of the puncture needle does not affect the success rate of renal puncture, which is more likely to be related to the puncture site. Therefore, the puncture success rate of the 18G needle is not different from that of the 16G needle.

Luciano [6] describes bleeding that requires blood transfusion, gross hematuria, arteriovenous fistula formation, and perirenal hematoma as the complications that occur after renal puncture. In our study, we found that the incidence of arteriovenous fistula, abdominal pain, gross hematuria, bleeding requiring blood transfusion, surgical intervention embolization, and renal resection was low, and there was no statistically significant difference between the two groups. The main complication was the formation of large hematomas, which was significantly different between the two groups (*P* = 0.003): 151 patients (5.98%) in the 18G needle group and 57 patients (9.31%) in the 16G needle group developed large hematomas.

With regard to factors that affect the size of hematomas that form after needle biopsy, previous research has shown that old age, hypertension, anemia, low platelet count, and kidney dysfunction are related to the size of the hematoma [23]. In this study, hematoma volume was significantly related to platelet count, serum creatinine, and cystatin C, but it was not significantly correlated with age, gender, BMI, hemoglobin level, 24-h urine protein level, GFR, or renal size (long diameter). The 18G group was significantly older than the 16G group and had significantly poorer renal function. Based on the previous findings, this would imply that the hematoma size was significant larger in the 18G group than in the 16G group. However, this was not the case based on the present findings. Therefore, our findings imply that the 18G needle can reduce the incidence of large hematomas, irrespective of patient age or renal status.

In previous studies, the incidence of gross hematuria following kidney biopsy has ranged from 3 to 9%, with blood transfusion being required in 0.1%–3% patients [21, 22]. In our sample, the incidence rate of gross hematuria was low (0.058%), and blood transfusion was needed in only 1/3138 (0.032%) patient; there was no statistically significant difference between the two groups. The low incidence may be because we used stent-guided puncture and sampled from the lateral segment of the kidney parenchyma, avoiding the renal medulla.

This study has some limitations. First, this was a single-center retrospective study and so bias is inevitable. Second, the 18G needle was solely used in the latter part of the study; the low incidence of complications may be partly attributable to the accumulated experience of the persons performing the biopsy.

To conclude, the 18G needle appears to be as effective as the 16G for percutaneous renal biopsy, and it may be less likely to cause complication. Our findings need to be confirmed in randomized clinical trials.

## Data Availability

The datasets used and/or analyzed during the current study are available from the corresponding author on reasonable request.

## Abbreviations

PRB: Percutaneous renal biopsy
BMI: Body mass index
GFR: Glomerular filtration rate
IQR: Interquartile range; SD, standard deviation
